# Supplementary material for: An Integrated Clinical-Biological Approach to Identify Interindividual Variability and Atypical Phenotype-Genotype Correlations in Myopathies: Experience on A Cohort of 156 Families
Source: Genes (Basel). 2021 Jul 31;12(8):1199. doi: 10.3390/genes12081199 (PMC8392536; doi:10.3390/genes12081199)
Supplement: Supplementary file 1 [file genes-12-01199-s001.zip › MORALES et al., Supplemental table 1.pdf]

Table S1: Extended gene panel (n=185) used for NGS study

**Gene list corresponding to the main clinical entry groups**	**Additional genes associated to other entry diagnostic categories included in the panel**
- Nemaline Congenital myopathies<br>- Congenital myopathies excluding nemaline associated genes<br>- Distal myopathies<br>- Myofibrillar myopathies and inclusion body myopathies<br>- Limb-Girdle Muscular Dystrophy<br>- Congenital Muscular Dystrophies except for alpha-dystroglycanopathies	- Alpha-dystroglycanopathies<br>- Metabolic myopathies<br>- Myopathies associated with joint contractures<br>- Congenital myasthenias<br>- Non-dystrophic myotonia<br>- Periodic paralysis<br>- Fetal and neonatal arthrogryposes<br>- Other myopathies
(official gene symbols approved by the HGNC) in red genes added during NGS panel update from 135 to 185 genes.	
ACAD9	*ABHD5*
ACTA1	*ACADVL*
ADSSL1	*ADGRG1*
ANO5	*AGL*
BAG3	*AGRN*
BIN1	*ALG14*
BVES	*ALG13*
CACNA1S	*ALG2*
CAPN3	*AMPD1*
CASQ1	*ATP2A1*
CAV3	*B3GALNT2*
CCDC78	*B4GAT1*
CFL2	*CACNA1A*
CHKB	*CAVIN1*
CNTN1	*CHAT*
COL12A1	*CHRNA1*
COL6A1	*CHRNB1*
COL6A2	*CHRND*
COL6A3	*CHRNE*
CRYAB	*CHRNG*
DAG1	*CLCN1*
DES	*CLN3*
DMD	*COL4A1*
DNAJB6	*COL13A1*
DNM2	*COLQ*

DPM3	*CPT2*
DYSF	*DOK7*
FLAD1	*DOLK*
FHL1	*DPAGT1*
FKRP	*DPM1*
FKTN	*DPM2*
FLNC	*ECEL1*
GAA	*EMD*
GMPPB	*ENO3*
GNE	*ETFA*
GOLGA2	*ETFB*
HACD1	*ETFDH*
HNRNPA1	*FBN2*
HNRNPDL	*GBE1*
ISPD	*GFPT1*
ITGA7	*GYG1*
KBTBD13	*GYS1*
KLHL40	*HSPG2*
KLHL41	*ISCU*
KLHL9	*KCNA1*
KY	*KCNE3*
LAMA2	*KCNJ18*
LDB3	*KCNJ2*
LIMS2	*LAMB2*
LMNA	*LAMP2*
LMOD3	*LDHA*
MATR3	*LARGE1*
MEGF10	*LARGE2*
MTM1	*LPIN1*
MYBPC3	*LRP4*
MYH2	*MUSK*
MYH7	*MYH3*
MYO18B	*MYH8*
MYOT	*MYO9A*
MYPN	*NALCN*
NEB	*NEK5*
ORAI1	*PABPN1*
PLEC	*PFKM*
PNPLA2	*PIEZO2*
POMGNT1	*PGAM2*
POMT1	*PGK1*
POMT2	*PGM1*
PYROXD1	*PHKA1*
RYR1	*PHKB*

SELENON	*PNPLA8*
SGCA	*POMGNT2*
SGCB	*POMK*
SGCD	*PREPL*
SGCG	*PRKAG2*
SPEG	*PYGM*
SQSTM1	*RAPSN*
STAC3	*RBCK1*
STIM1	*SCN4A*
TCAP	*SLC22A5*
TIA1	*SLC25A1*
TNNT1	*SLC25A20*
TNPO3	*SLC25A32*
TOR1AIP1	*SLC35A1*
TPM2	*SLC35A2*
TPM3	*SLC5A7*
TRAPPC11	*SNAP25*
TRIM32	*SYT2*
TRIM54	*TMEM5*
TRIM63	*TNNI2*
TRIP4	*TNNT3*
TTN	*TUBB2b*
VCP	*VMA21*
	ZC4H2
