# Supplementary material for: An Integrated Clinical-Biological Approach to Identify Interindividual Variability and Atypical Phenotype-Genotype Correlations in Myopathies: Experience on A Cohort of 156 Families
Source: Genes (Basel). 2021 Jul 31;12(8):1199. doi: 10.3390/genes12081199 (PMC8392536; doi:10.3390/genes12081199)
Supplement: Supplementary file 1 [file genes-12-01199-s001.zip › MORALES et al., Supplemental table 2.pdf]

Table S2: Detailed list of pathogenic, likely or possibly pathogenic variants

Patient	Gender, Age	Gene	Pathogenic variants	Variant type	Familial segregation	Familial data	ExAC/ gnomAD	SIFT/ Polyphen 2	MPA score	CADD	Splice AI	ACMG Class	Clinical features	CK	Muscle biopsy
I15	M, 4y	*ACTA1*<br>NM_001100.3	c.493G>C<br>p.(Val165Leu)	Missense	*De novo* variant	Sporadic	Absent	S : -<br>P : D<br>(0.93)	7.7	4,127	AG: 0 (-3)<br>AL: 0 (38)<br>DG: 0 (-8)<br>DL: 0 (1)	5	Severe neonatal hypotonia with respiratory failure	x2	Non-specific myopathic changes
I2	M, <1y	*ACTA1*	c.449C>T<br>p.(Thr150Ile)	Missense	Parents not tested	Sporadic	Absent	S : -<br>P : B<br>(0.38)	9	3,368	AG: 0 (31)<br>AL: 0 (48)<br>DG: 0 (-5)<br>DL: 0 (48)	4	Severe neonatal hypotonia with arthrogryposis	Nl	Non-specific myopathic changes
I100	M, <1y	*ACTA1*	c.1057A>G<br>p.(Thr353Ala)	Missense	Parents not tested	Sporadic	Absent	S : -<br>P : D<br>(0.91)	9	3,93	AG: 0 (36)<br>AL: 0 (-12)<br>DG: 0 (5)<br>DL: 0 (37)	4	Severe neonatal hypotonia with respiratory failure	Nl	Non-specific myopathic changes
I135	M, <1y	*ACTA1*	c.553C>T<br>p.(Arg185Cys)	Missense	*De novo* variant	Sporadic	Absent	S : -<br>P : D<br>(0.99)	10	4,574	AG: 0 (-44)<br>AL: 0 (-27)<br>DG: 0 (2)<br>DL: 0 (-35)	5	Severe neonatal hypotonia	x2	Non-specific myopathic changes
I192	M, 20y	*ACTA1*	c.889G>A<br>p.(Ala297Thr)	Missense	*De novo* variant	Sporadic	Absent	S : -<br>P : B<br>(0.07)	6.6	2,541	AG: 0 (9)<br>AL: 0 (40)<br>DG: 0 (10)<br>DL: 0 (5)	5	Asymptomatic CK elevation	x20	Non-specific myopathic changes
I32	F, 40y	*ANO5*<br>NM_213599.2	c.1622_1623insA<br>p.(Met543AsnFs*)	Frameshift indel	Mother was heterozygous	AR<br>(one affected sister)	4.37e-05/<br>3.68e-05		10	5,605	AG: 0 (7)<br>AL: 0 (-17)<br>DG: 0 (6)<br>DL: 0.25 (7)	5	Exercise intolerance	x20	Dystrophic pattern
			c.1991T>C<br>p.(Phe664ser)	Missense	Father not tested		Absent	S : T (0.1)<br>P : D<br>(0.97)	6	3,034	AG: 0 (-4)<br>AL: 0 (-2)<br>DG: 0 (38)<br>DL: 0 (-39)	3			
I53	M, 51y	*ANO5*	c.656A>G (homozygous)<br>p.(Tyr219Cys)	Missense	Not tested	Sporadic. Familial consanguinity	8.309e-06/<br>7.09e-06	S : D<br>(0.02)<br>P : D<br>(0.99)	9	4,013	AG: 0 (-7)<br>AL: 0 (13)<br>DG: 0.01 (45)<br>DL: 0 (-7)	4	LGMD	x20	Dystrophic pattern. Normal IHC
I303	F, 30y	*CACNA1S*<br>NM_000069.2	c.5104C>T<br>p.(Arg1702*)	Nonsense	Father was heterozygous	Affected deceased brother	9.309e-05/<br>8.492e-05		10	6,754	AG: 0 (-23)<br>AL: 0 (40)<br>DG: 0 (2)<br>DL: 0.02 (-30)	5	Neonatal hypotonia	Nl	Non-specific myopathic changes
			c.2970G>A<br>p.(Trp990*)	Nonsense	Mother was heterozygous		Absent		10	9,326	AG: 0 (-9)<br>AL: 0 (41)<br>DG: 0.79 (4)<br>DL: 0 (0)	5			
I164	M,20y	*CACNA1S*	c.2447T>G<br>p.(Leu816Arg)	Missense	Present in his pauci-symptomatic father	AD	Absent	S : D (0.0)<br>P : D<br>(0.99)	10	3,232	AG: 0 (50)<br>AL: 0 (8)<br>DG: 0 (-40)<br>DL: 0 (-43)	4	Neonatal hypotonia	Nl	Non-specific myopathic changes
I43	F, 36y	*CAPN3*<br>NM_000070.2	c.1250C>T<br>p.(Thr417Met)	Missense	Parents not tested	Sporadic	4.95e-05/<br>4,95e-05	S : D<br>(0.017)<br>P : D<br>(0.99)	9	4,084	AG: 0 (17)<br>AL: 0 (-19)<br>DG: 0 (40)<br>DL: 0 (42)	4	LGMD	x20	Dystrophic pattern. Normal IHC
			c.1536+3A>G	Splicing			Absent		10	2,852	AG: 0.04 (-14)<br>AL: 0 (-3)<br>DG: 0 (-15)<br>DL: 0.27 (-3)	4			

I219	M, 23 y	*CAPN3*	c.1469G>A (homozygous) p.(Arg490Gln)	Missense	Not tested	Sporadic	5.95e-05/ 6,725e-05	S : D (0.01) P : D (1)	8	4,12	AG: 0 (2) AL: 0 (-32) DG: 0 (-44) DL: 0 (19)	4	LGMD	x10	Dystrophic pattern. Normal IHC
I144, I148	F,9y M, 40y	*COL6A1* NM_001848 .2	c.299_428+152delinsGA CAGGA	CNV	Affected brother and father were heterozygous	AD	Absent	S: D (0) P: D (0.99)	10	NA	NA	5	CM with early joint contractures	x2	Non-specific myopathic changes
I269	F, 36y	*COL6A1*	c.806G>A p.(Gly269Glu)	Missense	*De novo* variant	Sporadic	Absent	S : D (0) P : D (0.99)	10	4,685	AG: 0.55 (3) AL: 0 (9) DG: 0 (3) DL: 0 (8)	4	CM with contractures and respiratory failure	UNK	Dystrophic pattern
I65	M, 10y	*COL6A1*	c.739-1G>A	Splicing	Present in affected father	AD	Absent		10	5,196	AG: 0.48 (22) AL: 1 (1) DG: 0 (-50) DL: 0 (21)	5	CM with contractures	Nl	Not done
I93	M, 10y	*COL6A2* NM_001849 .3	c.403G>A p.(Asp135Asn)	Missense	Present in mother	Sporadic	Absent/ 4e-06	S : D (0) P : D (0.98)	10	4,049	AG: 0 (30) AL: 0 (-33) DG: 0 (3) DL: 0 (22)	4	LGMD with contractures	x5	Dystrophic pattern
			c.2905G>A p.(Val969Met)	Missense	Present in father		1.702e-05/ 2e-06	S : D (0.007) P : D (0.983)	9	3,592	AG: 0 (29) AL: 0 (-3) DG: 0 (2) DL: 0 (-1)	4			
I429	M, 20y	*COL6A3* NM_004369 .3	c.6170A>G p.(Glu2057Gly)	Missense	Not tested	Sporadic	Absent	S : T (0.082) P : D (0.974)	8	2,928	AG: 0 (36) AL: 0 (13) DG: 0 (-44) DL: 0 (-40)	4	LGMD	x3	Not done
I542	M, 4y	*COL6A3*	c.6230G>T p.(Gly2077Val)	Missense	*De novo* variant	Sporadic	Absent	S : D (0) P : D (1)	10	4,371	AG: 0 (-18) AL: 0 (49) DG: 0 (19) DL: 0 (1)	4	CM	Nl	Non-specific myopathic changes
I58	F, 35y	*COL6A3*	c.5035G>T p.(Gly1679Trp)	Missense	Present in affected mother, uncle and cousin	AD	Absent	S : D (0) P : D (1)	10	4,116	AG: 0 (-12) AL: 0 (-48) DG: 0 (2) DL: 0 (4)	4	LGMD with retractions		Non-specific myopathic changes
I287	F, 16y	*DMD* NM_004006 .2	del <45-51> (heterozygous)	CNV : Deletion	Present in mother	Sporadic				NA	NA	5	Asymptomatic elevation of CK	x10	Not done
I56	F, 45Y	*DMD*	c.2665C>T p.(Arg889*)	Nonsense	Mother was heterozygous	X-linked The mother had high CK	Absent		10	6,71	AG: 0 (49) AL: 0.01 (42) DG: 0 (42) DL: 0 (4)	5	LGMD	x10	Dystrophic pattern. IHC studies not done
I12	F, 61 y	*DYSF* NM_003494 .3	c.1168G>A p.(Asp390Asn)	Missense	Affected brother carrying the same variants. Parents not tested	AR	Absent/ 7.981e-06	S : D (0) P : D (0.9)	8	3,87	AG: 0 (-16) AL: 0 (-50) DG: 0.08 (-16) DL: 0 (33)	4	LGMD	x10	Dystrophic pattern. IHC: Absence of dysferlin staining
			c.5302C>T p.(Arg1768Trp)	Missense			10.62e-06/ 8.283e-06	S : D (0) P : D (1)	9	4,429	AG: 0 (14) AL: 0 (-30) DG: 0 (42) DL: 0 (38)	4			
I16	M, 68y	*DYSF*	c.755C>T p.(Thr252Met)	Missense	Parents not tested	AR (one affected sister)	Absent/ 11.93e-06	S : D (0) P : D (1)	10	3,583	AG: 0 (-28) AL: 0 (-15) DG: 0 (37) DL: 0 (40)	4	LGMD	x2	Dystrophic pattern. IHC of dysferlin on muscle biopsy not done
			c.3118C>T p.(Arg1040Trp)	Missense			Absent	S : D (0) P : D (0.99)	10	4,146	AG: 0 (-10) AL: 0 (-48) DG: 0 (-20) DL: 0 (8)	4			
I111	F, 4y	*FLNC* NM_001458 .4	c.4927+2T>A	Splicing	*De novo* variant	Sporadic	Absent		10	5,384	AG: 0 (2) AL: 0 (-18) DG: 0.03 (2) DL: 0.99 (-2)	4	CM with restrictive cardiomyopathy	Nl	Non-specific myopathic changes

I298	M, 38y	*FLNC*	c.3557C>T<br>p.(Ala1186Val)	Missense	*De novo* variant	Sporadic	Absent	S : D (0.001)<br>P : D (0.8)	9	3,452	AG: 0 (41)<br>AL: 0 (-12)<br>DG: 0.01 (-50)<br>DL: 0 (46)	4	Myopathy with contractures and early onset cardiomyopathy	UNK	Cytoplasmic bodies
I202	M, <1y	*FKRP*<br>NM_024301.4	c.1364C>A (homozygous)<br>p.(Ala455Asp)	Missense	Mother and father were heterozygous	Sporadic (Consanguinity)	Absent	S : D (0.005)<br>P : D (0.898)	8	4,068	AG: 0 (17)<br>AL: 0 (47)<br>DG: 0 (16)<br>DL: 0 (2)	4	CMD with cerebellar atrophy	x50	Not done
I218	M, 50y	*FKRP*	c.826C>A (homozygous)<br>p.(Leu276Ile)	Missense	Not tested	Sporadic	0,003961/0.001104	S : T (0.065)<br>P : B (0.3)	5	2,015	AG: 0 (4)<br>AL: 0 (16)<br>DG: 0 (2)<br>DL: 0 (-9)	4	Severe LGMD	UNK	Dystrophic pattern. IHC not done
I354	F, <1y	*FKRP*	c.1384C>T (homozygous)<br>p.(Pro462Ser)	Missense	Mother and father were heterozygous	Sporadic Familial consanguinity	1.448e-05/<br>9.037e-06	S : D (0)<br>P : D (0.996)	10	3,891	AG: 0 (27)<br>AL: 0 (-3)<br>DG: 0 (-2)<br>DL: 0 (-4)	4	CMD	x30	Dystrophic pattern. Absence of α-dystroglycan staining
I24	M, 40y	*GMPPB*<br>NM_013334.3	c.902C>G<br>p.(Ser301Cys)	Missense	Mother not tested	Sporadic	Absent	S : D (0)<br>P : B (0.152)	7	2,304	AG: 0 (32)<br>AL: 0 (-5)<br>DG: 0 (-37)<br>DL: 0 (-41)	4	LGMD	x10	Dystrophic pattern
			c.1069G>A<br>p.(Val357Ile)	Missense	Present in father		7.438e-05/<br>6.017e-05	S: D (0)<br>P: D (1)	8	3,857	AG: 0 (-38)<br>AL: 0.01 (36)<br>DG: 0.01 (9)<br>DL: 0 (-38)	4			
I252	F, <1y	*LAMA2*<br>NM_000426.3	c.1884+1_1884+6del (homozygous)	Frameshift indel	Not tested	Sporadic	Absent		10	NA	NA	5	Severe neonatal hypotonia	x20	Not done
I29	M, 17y	*LAMA2*	c.752T>C (homozygous)<br>p.(Leu251Pro)	Missense	Parents was heterozygous	Sporadic	Absent	S : D (0)<br>P : D (0.998)	10	4,006	AG: 0 (-13)<br>AL: 0 (33)<br>DG: 0 (49)<br>DL: 0 (-34)	4	CMD, intellectual deficiency and epilepsy	x50	Dystrophic pattern. Absence of α-dystroglycan staining
I30	M, 28y	*LAMA2*	c.2461A>C<br>p.(Thr821Pro)	Missense	Mother was heterozygous	Sporadic	Absent	S : D (0.002)<br>P : D (0.998)	10	3,853	AG: 0 (-10)<br>AL: 0 (-18)<br>DG: 0 (-4)<br>DL: 0 (-19)	4	LGMD	x10	Dystrophic pattern. Normal IHC
			c.3976C>T<br>p.(Arg1326*)	Nonsense	Father was heterozygous		Absent		10	7,952	AG: 0 (-10)<br>AL: 0 (-47)<br>DG: 0 (-47)<br>DL: 0 (15)	5			
I20	F, 2y	*LMNA*<br>NM_170707.3	c.1357C>T<br>p.(Arg453Trp)	Missense	*De novo* variant	Sporadic	Absent	S : D (0)<br>P : D (0.993)	9	4,613	AG: 0 (40)<br>AL: 0 (-46)<br>DG: 0 (-25)<br>DL: 0 (23)	5	CMD	x10	Dystrophic pattern
I207	M, <1y	*MTM1*<br>NM_000252.2	c.290G>A<br>p.(Gly97Glu)	Missense	Mother was heterozygous	Sporadic	Absent	S : D (0.001)<br>P : D (0.999)	10	3,897	AG: 0 (-14)<br>AL: 0 (16)<br>DG: 0 (47)<br>DL: 0 (-28)	4	Severe neonatal hypotonia with Respiratory failure	Nl	Not done
I7	M, <1y	*MTM1*	c.519C>G<br>p.(Tyr173*)	Nonsense	Not tested	Sporadic	Absent		10	5,834	AG: 0 (1)<br>AL: 0 (-49)<br>DG: 0.01 (5)<br>DL: 0 (9)	5	Severe neonatal hypotonia with respiratory failure	Nl	Centronuclear myopathy
I40	F, 48y	*MTM1*	c.1261-10A>G (ivs11)	Splicing	Sister affected	X-Linked	Absent		10	3,022	AG: 0.93 (1)<br>AL: 0.86 (10)<br>DG: 0 (1)<br>DL: 0 (17)	4	LGMD	x2	Non-specific myogenic changes

I195	F, 56y	*MYH7* NM_000257.2	c.5791-1G>T	Splicing	Present in mother	Sporadic (the clinical examination of the mother was not available)	Absent		10	5,392	AG: 0.22 (29) AL: 0.95 (-1) DG: 0 (-13) DL: 0 (-9)	4	Early onset cardiomyopathy and LGMD of adult onset	UNK	Minicores
I208	M, 4y	*MYH7*	c.5000T>C p.(Leu1667Pro)	Missense	*De novo* variant	Sporadic	Absent	S : D (0) P : D (0.94)	10	4,225	AG: 0 (18) AL: 0 (-47) DG: 0 (-1) DL: 0 (-4)	4	CM	Nl	Minicores
I36, I37	M, 40y	*MYH7*	c.4795A>C p.(Thr1599Pro)	Missense	Present in affected daughter	AD	Absent	S : D (0.001) P : P (0653)	10	3,461	AG: 0 (-16) AL: 0 (0) DG: 0 (19) DL: 0 (-44)	4	Distal myopathy	x5	Non-specific myogenic pattern IHC study: normal
I62	F, 50y	*MYOT* NM_006790	c.179C>G p.(Ser60Cys)	Missense	Present in affected brother	Affected brother	Absent	S : D (0.004) P : D (0.894)	5	3,687	AG: 0 (-49) AL: 0 (47) DG: 0 (-1) DL: 0 (-40)	4	Distal myopathy	x2	Non-specific myogenic pattern IHC study: normal
I353	M, 19y	*NEB* NM_001271208.1	c.2835+5G>A	Splicing	Present in mother	Sporadic	Absent		10	3,159	AG: 0 (-19) AL: 0 (25) DG: 0 (26) DL: 0.83 (5)	4	CM	Nl	Rods
			c.15178delC p.(His5060Thrfs*11)	Frameshift	Father not tested		Absent		10	NA	AG: 0.02 (-24) AL: 0 (39) DG: 0.05 (45) DL: 0 (39)	5			
I76, I77	M, 54y F, 50y	*NEB*	c.21928T>C p.(Ser7310Pro)	Missense	Father not tested	AR (one affected sister)	Absent	S : T (0.114) P : P (0.653)	3	3,876	AG: 0 (-43) AL: 0 (12) DG: 0.01 (-43) DL: 0 (-17)	4	Distal myopathy	Nl	Non-specific myopathic changes
			c.8860del p.(Ala2954Profs*8)	Frameshift	Mother was heterozygous		Absent		10	NA	AG: 0 (-29) AL: 0 (28) DG: 0 (-6) DL: 0.04 (-29)	5			
I173	F, 16y	*NEB*	c.5039G>A p.(Tyr1680Cys)	Missense	Father was heterozygous	Sporadic	Absent	S : D (0) P : D (0.999)	7	4,135	AG: 0.02 (-30) AL: 0 (7) DG: 0 (-21) DL: 0 (3)	3	CM	Nl	Rods
			c.17541dupA p.(Tyr5848Ilefs*15)	Frameshift	Mother was Heterozygous		Absent		10	6,815	AG: 0 (5) AL: 0 (23) DG: 0 (0) DL: 0 (1)	5			
I112	M, 60y	*NEB*	c.21790G>C p.(Asp7264His)	Missense	Parents not tested	Sporadic	0.001906/0.001829	S : D (0.001) P : D (0.897)	6	4,263	AG: 0 (-22) AL: 0 (-1) DG: 0 (-38) DL: 0 (-50)	4	Distal myopathy	Nl	Non-specific myopathic changes
			c.194C>T p.(Pro65Leu)	Missense			14.12e-05/11.77e-05	S : D (0) P : B (0.32)	4	2,865	AG: 0 (1) AL: 0 (42) DG: 0 (1) DL: 0 (-16)	3			
I1	M, <1y	*NEB*	c.6075+5G>A	Splicing	Present in father	Sporadic	Absent		10	4,228	AG: 0 (-34) AL: 0 (-6) DG: 0.92 (-46) DL: 0.96 (5)	4	Severe neonatal hypotonia and distal arthrogryposis.	Nl	Rods
			c.13661_13666delinsA p. (Ser4554Asnfs*10)	Frameshift	Present in mother		Absent		10	NA	NA	5			
I67	F, 1y	*NEB*	c.23998dupG p.(Glu8000Glyfs*11)	Frameshift	Father was heterozygous	Sporadic	Absent		10	NA	AG: 0 (44) AL: 0 (-41) DG: 0 (-26) DL: 0.16 (-35)	4	Severe neonatal hypotonia with	Nl	Non-specific myopathic changes

			c.518delA p.(Lys173Serfs*55)	Frameshift	Mother was heterozygous		Absent		10	NA	AG: 0.02 (-2)<br>AL: 0 (46)<br>DG: 0 (-2)<br>DL: 0 (11)	5	respiratory failure.		
I604	M, 7Y	*NEB*	c.8889+1G>A	Splicing	Father was heterozygous	Sporadic	Absent		10	5,898	AG: 0 (0)<br>AL: 0 (35)<br>DG: 0 (-25)<br>DL: 0.99 (1)	4	Severe neonatal hypotonia with respiratory failure and arthrogryposis	x2	Rods
			c.8038C>T p.(Arg2680*)	Nonsense	Mother was heterozygous		Absent		10	7,495	AG: 0 (-39)<br>AL: 0 (15)<br>DG: 0 (-33)<br>DL: 0 (-13)	5			
I14	F, 75y	*PABPN1* NM_004643.3	c.30_32dup p.(Ala11dup)	Trinucleotid repeat expansion	Not tested	Sporadic				2,144	NA	4	LGMD with axial weakness	Nl	Myofibrillar aspect with rimmed vacuoles
I5	M, <1y	*RYR1* NM_000540.2	c.11186T>C p.(Met3729Thr)	Missense	Parents not tested	Sporadic	Absent	S : D (0.021)<br>P : D (0.946)	9	3,832	AG: 0 (-44)<br>AL: 0 (8)<br>DG: 0 (7)<br>DL: 0 (-24)	4	Distal arthrogryposis. Neonatal death	Nl	Cores
			c.11122A>C p.(Thr3708Pro)	Missense			Absent	S : T (0.157)<br>P : P (0.876)	7	4	AG: 0 (19)<br>AL: 0 (-35)<br>DG: 0 (-23)<br>DL: 0 (19)	4			
I42	F, 11y	*RYR1*	c.38T>C p.(Leu13Pro)	Missense	Mother was heterozygous	Sporadic	Absent	S : D (0.023)<br>P : D (1)	9	4,104	AG: 0 (7)<br>AL: 0 (-24)<br>DG: 0 (-11)<br>DL: 0 (7)	4	CM	Nl	Non-specific myopathic changes
			c.7628C>T p.(Thr2543Ile)	Missense	Mother was heterozygous		Absent/ 3.190 e-05	S : T (0.09)<br>P : P (0.506)	6	2,996	AG: 0.06 (-2)<br>AL: 0 (-8)<br>DG: 0 (-2)<br>DL: 0 (1)	3			
			c.13690C>T p.(Arg4564Trp)	Missense	Father was heterozygous		4.118e-05/ 3.579e-05	S : D (0)<br>P : D (0.997)	9	4,097	AG: 0 (-24)<br>AL: 0 (-30)<br>DG: 0 (-2)<br>DL: 0 (46)	3			
I220	M, 3y	*RYR1*	c.14762T>C p.(Phe4921Ser)	Missense	*De novo* variant	Sporadic	Absent	S : D (0)<br>P : D (0.994)	9	4,335	AG: 0 (-5)<br>AL: 0 (-20)<br>DG: 0 (-43)<br>DL: 0 (-20)	5	CM	Nl	Non-specific myopathic changes
I175	F,10y	*RYR1*	c.12727G>A p.(Glu4243Lys)	Missense	*De novo* variant	Sporadic	Absent	S : D (0.02)<br>P : D (1)	9	4,183	AG: 0 (3)<br>AL: 0 (9)<br>DG: 0 (8)<br>DL: 0 (-28)	4	CM	Nl	Non-specific myopathic changes
I176	M, <1y	*RYR1*	c.5926C>T p.(Arg1976Cys)	Missense	Present in mother	Sporadic	Absent	S : D (0)<br>P : D (1)	8	3,999	AG: 0 (22)<br>AL: 0 (-6)<br>DG: 0 (-2)<br>DL: 0 (2)	4	Severe neonatal hypotonia	Nl	Non-specific myopathic changes
			c.10649G>C p.(Arg3550Pro)	Missense	Present in father		Absent	S : D (0)<br>P : D (1)	8	3,809	AG: 0 (-22)<br>AL: 0 (19)<br>DG: 0.03 (37)<br>DL: 0 (-5)	4			
I155	F, 51y	*RYR1*	c.6697T>C p.(Cys2233Arg)	Missense	Parents not tested	AD (affected son)	Absent	S : D (0.001)<br>P : D (0.996)	9	0,025	AG: 0 (1)<br>AL: 0 (-37)<br>DG: 0 (14)<br>DL: 0 (-23)	5	CM	Nl	Nl
I519	F, 26y	*RYR1*	c.10348-6C>G	Splicing	Father not tested	Sporadic	2.475e-05/ 3.182e-05		10	0,45	AG: 0 (-41)<br>AL: 0.47 (6)<br>DG: 0 (35)<br>DL: 0 (15)	5	CM	Nl	Non-specific myopathic changes
			c.14172+4A>G	Splicing	Present in mother		8.245e-06/ 7.958e-06		10	2,34	AG: 0 (50)<br>AL: 0 (-46)<br>DG: 0.01 (-20)<br>DL: 0.08 (-4)	4			

I526	M, 8y	*RYR1*	c.1205T>C p.(Met402Thr)	Missense	Mother was heterozygous	Sporadic	8.245e-06/ 3.97e-06	S : D (0.003) P : P (0.73)	8	2,684	AG: 0 (1) AL: 0 (10) DG: 0 (-4) DL: 0 (39)	5	CM	Nl	Minicores
			c.10496dupG p.(Arg3499fs)	Frameshift delins	Father not tested		Absent/ 4.06e-06		10	5,692	AG: 0 (23) AL: 0 (-22) DG: 0 (9) DL: 0 (-22)	5			
I152	M, 35 y	*RYR1*	c.6617C>T (homozygous) p.(Thr2206Met)	Missense	Parents not tested	Affected sister	3.305e-05/ 2.123e-05	S : D (0) P : D (0.998)	9	3,864	AG: 0 (-1) AL: 0 (-48) DG: 0 (35) DL: 0 (10)	4	LGMD	x20	Non-specific myopathic changes
I46	M, 60 y	*RYR1*	c.4711A>G p.(Ile1571Val)	Missense	Parents not tested	Sporadic	0.001339/8. 548e-04	S : T (0.729) P : D (0.978)	6	1,318	AG: 0 (-3) AL: 0 (16) DG: 0 (30) DL: 0 (10)	4	Proximal and distal weakness	UNK	Myofibrillar aspects
			c.10097G>A p.(Arg3366His)	Missense			9.272e-04/ 8.605e-04	S : D (0.006) P : D (0.997)	7	2,753	AG: 0.01 (5) AL: 0 (17) DG: 0 (7) DL: 0 (-5)	4			
			c.11798A>G p.(Tyr3933Cys)	Missense			9.233e-04/ 8,487e-04	S : D (0) P : D (0.997)	9	4,166	AG: 0 (-50) AL: 0 (-8) DG: 0 (14) DL: 0 (3)	4			
			c.7028-10G>A	Splicing			Absent/ 1,864e-05		10	2,885	AG: 1 (2) AL: 0.97 (10) DG: 0 (41) DL: 0 (9)	5			
I28	F, 14y	*SGCA* NM_000023 .2	c.850C>T (homozygous) p.(Arg284Cys)	Missense	Mother and father were heterozygous	AR Consangui nity (Affected sister)	1.237e-04/ 1.485e-04	S : D (0) P : D (0.995)	9	4,412	AG: 0 (-13) AL: 0 (-9) DG: 0 (0) DL: 0 (11)		Exercise intolerance	x40	Dystrophic pattern. Normal IHC
I31	M, 15y	*SGCG* NM_000231 .2	c.800_801del (homozygous) p.(Cys267Serfs*51)	Frameshift	Parents were heterozygous	Sporadic	Absent		10	4,452	AG: 0 (-22) AL: 0 (13) DG: 0 (41) DL: 0 (0)	5	LGMD	x50	Dystrophic pattern. Absence of γ-sarcoglycan staining
I427	M, 20y	*STIM1* NM_003156 .3	c.910C>T p.(Arg304Trp*)	Nonsense	Not tested	Sporadic	Absent	S : D (0.001) P : D (0.998)	8	3,912	AG: 0 (15) AL: 0 (-24) DG: 0 (-2) DL: 0 (6)	5	Features of Stormorken syndrome: myalgia, headaches, myosis	x30	Tubular aggregates
I26	M, 14y	*TTN* NM_001267 550.1	c.51437-4_51444del	Frameshift delins	Not feasible (adopted child)	Sporadic	Absent		10	NA	NA	4	CM	Nl	Centronucle ar myopathy
			c.26503A>T p.(Lys8835*)	Nonsense			Absent		10	9,482	AG: 0 (32) AL: 0.02 (20) DG: 0 (20) DL: 0 (-48)	5			
I398	F, 10y	*TTN*	c.56648-1G>A	Splicing	Mother was heterozygous	Sporadic	9.008e-06/ 5.321e-06		10	5,739	AG: 0.11 (-19) AL: 0.99 (-1) DG: 0 (-13) DL: 0 (-38)	4	CM with arthrogryposis and rigid spine	Nl	Cores
			c.40011delA p.(Lys13337fs)	Frameshift	Father was heterozygous		Absent		10	NA	AG: 0 (37) AL: 0 (38) DG: 0.1 (-46) DL: 0 (9)	5			

I47	F, 44y	*TTN*	c.65575+2T>G	Splicing	Mother was heterozygous, father not tested	Sporadic	Absent		10	5,014	AG: 0 (7)<br>AL: 0 (22)<br>DG: 0.02 (-30)<br>DL: 1 (2)	5	CM	Nl	Excessive internal nuclei.
			c.68029A>G p.(Thr22677Ala)	Missense Predicted to affect splicing	Absent in mother, father not tested		Absent	S: D (0.012)<br>P: P (0.622)	4.4	2,467	AG: 0 (-16)<br>AL: 0 (-18)<br>DG: 0 (-40)<br>DL: 0 (38)	4			
142, I162	F, 40y	*TTN*	c.1662+15_3101-3del	CNV	Affected mother was heterozygous	AD	Absent			NA	NA	5	Distal Myopathy	x2	Minicores
I420	M, 56y	*TTN*	c.35713+1del	Splicing	Not tested	Affected brother and sister	5.58 e-05		10	5,074	AG: 0 (-24)<br>AL: 0 (22)<br>DG: 0.08 (2)<br>DL: 0.98 (1)	4	LGMD with cardiomyopath y	x5	Myofibrillar features
			c.7120C>T p.(Gln2374*)	Nonsense			Absent		10	7,175	AG: 0 (-3)<br>AL: 0 (38)<br>DG: 0 (22)<br>DL: 0 (18)	5			
I74, I73	F, 4y	*TTN*	c.106531G>C; p.(Ala35511Pro)	Missense (predicted to affect splicing)	Present in father	AR (one affected sister)	Absent	UNK	10	5,054	AG: 0 (0)<br>AL: 0 (50)<br>DG: 0 (-4)<br>DL: 0.62 (0)	5	CM	Nl	Centronucle ar myopathy
			c.105036C>A p.(Tyr35012*)	Nonsense	Present in mother		Absent		10	15,61	AG: 0 (-25)<br>AL: 0 (9)<br>DG: 0 (-25)<br>DL: 0 (-6)	5			
I401	F, 1y	*TNNT1* NM_003283 .5	c.192+244_388-1191del (del<8-9>) (homozygous)	In Frame CNV deletion	Father and mother heterozygous	Sporadic	Absent		10	NA	NA	4	Severe neonatal hypotonia	Nl	Rods
I72	F, 11y	*TNNT1*	c.200A>G p.(His67Arg)	Missense	*De novo* variant	Sporadic	Absent	S : D (0.002)<br>P : B (0.22)	7	3,721	AG: 0.01 (42)<br>AL: 0 (7)<br>DG: 0 (-49)<br>DL: 0 (-2)	4	Neonatal hypotonia	Nl	Excessive internal nuclei. Absence of rods
I60	M, 50y	*TRIP4*	del <8-9> (homozygous)	In Frame CNV deletion	Absent in two sister and one brother	Sporadic Consangui nity	Absent			NA	NA	4	Congenital myopathy with late onset cardiomyopath y	Nl	Rods

AD: Autosomal Dominant; AR: Autosomal Recessive; CM: Congenital myopathy; LGMD: Limb girdle muscular dystrophy; CK: Creatin Kinase; Unknown: UNK; ND: Not done; NA : Not Applicable; IHC: Immunohistochemical; S: SIFT; P: POLYPHEN2; CNV: Copy number variation; D: Deleterious; P: Pathogenic; B: Benign; T: Tolerated; Splice AI – AG: Acceptor Gain AL: Acceptor Loss DG: Donor Gain DL: Donor Loss (distance in bp)
